# Isolation and characterization of bacteriophages for combating multidrug-resistant *Listeria monocytogenes* from dairy cattle farms in conjugation with silver nanoparticles

**DOI:** 10.1186/s12866-023-02893-y

**Published:** 2023-05-22

**Authors:** Mona M. Elsayed, Rasha M. Elkenany, Amira I. Zakari, Basma M. Badawy

**Affiliations:** 1 Department of Hygiene and Zoonoses, Faculty of Veterinary Medicine, Mansoura University, Mansoura, 35516 Egypt; 2 Department of Bacteriology, Immunology, and Mycology, Faculty of Veterinary Medicine, Mansoura University, Mansoura, 35516 Egypt; 3 Department of Food Hygiene and Control, Faculty of Veterinary Medicine, Mansoura University, Mansoura, 35516 Egypt

**Keywords:** Bacteriophages, Dairy cattle farms, Time killing curve, *Listeria monocytogenes*, Multidrug- resistant, Silver nanoparticles

## Abstract

**Background:**

This study aims to achieve biocontrol of multidrug-resistant *Listeria monocytogenes* in dairy cattle farm**s** which poses a severe threat to our socio-economic balance and healthcare systems.

**Methods:**

Naturally occurring phages from dairy cattle environments were isolated and characterized, and the antimicrobial effect of isolated *L. monocytogenes* phages (LMPs) against multidrug-resistant *L. monocytogenes* strains were assessed alone and in conjugation with silver nanoparticles (AgNPs).

**Results:**

Six different phenotypic LMPs (LMP1–LMP6) were isolated from silage (*n* = 4; one by direct phage isolation and three by enrichment method) and manure (*n* = 2; both by enrichment method) from dairy cattle farms. The isolated phages were categorized into three different families by transmission electron microscopy (TEM): *Siphoviridae* (LMP1 and LMP5), *Myoviridae* (LMP2, LMP4, and LMP6), and *Podoviridae* (LMP3). The host range of the isolated LMPs was determined by the spot method using 22 multidrug-resistant *L. monocytogenes* strains. All 22 (100%) strains were susceptible to phage infection; 50% (3 out of 6) of the isolated phages showed narrow host ranges, while the other 50% showed moderate host ranges. We found that LMP3 (the phage with the shortest tail) had the ability to infect the widest range of *L. monocytogenes* strains. Eclipse and latent periods of LMP3 were 5 and 45 min, respectively. The burst size of LMP3 was 25 PFU per infected cell. LMP3 was stable with wide range of pH and temperature. In addition, time-kill curves of LMP3 alone at MOI of 10, 1 and 0.1, AgNPs alone, and LMP3 in combination with AgNPs against the most phage-resistant *L. monocytogenes* strain (ERIC A) were constructed. Among the five treatments, AgNPs alone had the lowest inhibition activity compared to LMP3 at a multiplicity of infection (MOI) of 0.1, 1, and 10. LMP3 at MOI of 0.1 in conjugation with AgNPs (10 µg/mL) exhibited complete inhibition activity after just 2 h, and the inhibition activity lasted for 24 h treatment. In contrast, the inhibition activity of AgNPs alone and phages alone, even at MOI of 10, stopped. Therefore, the combination of LMP3 and AgNPs enhanced the antimicrobial action and its stability and reduced the required concentrations of LMP3 and AgNPs, which would minimize the development of future resistance.

**Conclusions:**

The results suggested that the combination of LMP3 and AgNPs could be used as a powerful and ecofriendly antibacterial agent in the dairy cattle farm environment to overcome multidrug-resistant *L. monocytogenes*.

**Supplementary Information:**

The online version contains supplementary material available at 10.1186/s12866-023-02893-y.

## Background

*Listeria monocytogenes* is ubiquitous in the environment and can be isolated from water, soil, animal feed, industrial plants, and farms [1]. *L. monocytogenes* infection has the highest mortality rate of all food-borne infections, with a case fatality rate of 20%–30% [2]. Antibiotic resistance, which is becoming widespread in the veterinary field, is now considered a severe public health threat. The overuse and misuse of antibiotics have resulted in a high rate of multidrug resistance in food-borne pathogens in general, and in *L. monocytogenes* in particular, over the past two decades [3]*.* Multidrug resistance was recorded in 100% of *L. monocytogenes* isolates obtained from dairy cattle farms in two previous studies [4, 5]. Antibiotic resistance illnesses kill over 700,000 people each year around the world. By 2050, if no plan is implemented to prevent the spread of antimicrobial resistance, the predicted death rate is expected to rise to 10 million, resulting in economic losses of more than $100 trillion [6].

Alternatives to antibiotics are therefore required for treating various bacterial diseases. The use of lytic bacterial viruses (bacteriophages) as a biocontrol strategy is a promising alternative to antibiotics. Bacteriophages are viruses that infect and destroy their bacterial host cells. The interaction between the phage and its specific host ensures that pathogenic bacteria are targeted so that mammalian or human cells are not harmed [7]. In addition, the risk of secondary component formation has not been reported when using bacteriophages, in contrast to the use of chemical agents [8]. Although laboratory isolation and preparation of phages requires less effort, time, and money than the preparation of antibiotics, more work is required to produce phage products with long shelf lives for application in the field [9]. However, the development of bacterial resistance to phages could occur during the phage treatment process [10]. Recent approaches suggest that the conjugation of phages with biomaterials such as nanoparticles could improve phage tolerance, transport, and efficiency [11].

Nanotechnology has been investigated as a technique for delivering bacteriophages to specific locations in food or shielding them from harmful environmental conditions [12]. Nowadays, silver compounds are routinely applied in a wide array of industrial and sanitary fields, such as coating of catheters and surgery material, the production of synthetic compounds for odontology, treatment of burn injuries, homeopathic medicine or water purification [13, 14]. Silver nanoparticles (AgNPs) from 5 to 100 nm is more stable and absorbed at a much lower extent by eucaryotic cells, so it has minimal toxic effect [15], which explains why its use has been promoted in the last decades [14].

Recently, new methods have been established that use AgNPs as a carrier for bacteriophages. The formation of phage-metallic-nanoparticle networks (bio-nanotechnology) makes the phage more resistant to heat, pH, and organic solvents (such as 50% methanol); in addition, this method is inexpensive [16]. The use of phage-AgNP composites as a treatment after in vitro experimental studies will help us better understand the stability and interactions between phages and nanoparticles, as well as provide a possible biocontrol agent for a variety of applications. To our knowledge, no method for the biocontrol of multidrug-resistant *L. monocytogenes* isolated from dairy cattle farms has been reported using either phages or a combination of phages and AgNPs.

The aim of this study was to develop a new strategy for combating multidrug-resistant *L. monocytogenes* in the environment of dairy cattle farms via the isolation and characterization of naturally occurring phages from the dairy cattle environment and the detection of their host ranges. We evaluated the antimicrobial effects of the most efficient *L. monocytogenes* phage (LMP) alone and in conjugation with silver nanoparticles (LMP-AgNPs) to assess the potential synergistic impact for combating multidrug-resistant *L. monocytogenes* strains.

## Methods

### Samples collection

A total of 72 silage and manure samples (*n* = 36 each) were collected from three dairy cattle farms between February 2021 and June 2021. Silage samples were collected in a sterile Whirl–Pak bag (Nasco, Modesto, CA) [17]. An acidic pH (5.5) in silage indicates poor fermentation and an increased chance of bacteriophage isolation. Composite samples of manure were collected from different locations in various barns using sterile plastic sleeves. Permission to collect samples was obtained from the farms’ owners.

### Bacterial strains (hosts)

Multidrug-resistant *L. monocytogenes* referenced strains (*n* = 22) with different Enterobacterial Repetitive Intergenic Consensus (ERIC) sequences (A–V) were obtained from the Hygiene and Zoonosis Department at the Faculty of Veterinary Medicine of Mansoura University, Egypt, as indicated in (Supplementary Table 1) [5]. Each strain (0.1 mL) was refreshed in 10 mL of brain heart infusion (BHI) broth (Oxoid TM, Hampshire, UK) and incubated for 16 h at 30 °C. The concentrations were adjusted to OD600 which corresponded to 3 × 10^7^ colony-forming units (CFU)/mL using a spectrophotometer (Automatic Elisa Plate Analyser, Robonik, India).

### *Listeria monocytogenes* phage (LMP) isolation

Isolation of LMPs from silage and manure samples was performed by two different methods: direct isolation and isolation after enrichment.

- **a) Direct isolation method:** A 10 g sample of silage or manure was homogenized manually in 90 mL of phosphate-buffered saline (PBS) in a sterile Whirl–Pak bag with a filtered screen. Then, each mixture was filtered, first through a bottle-top filter with a pore size of 0.45 μm, and then through a syringe filter with a pore size of 0.2 μm [18]. The filtrate for each sample was used for phage isolation using the double-layer plate method. In brief, 300 µL of the previously prepared *L. monocytogenes* strain (approximately 3 × 10^7^ CFU/mL) was mixed with the sample filtrate (100 µL) and 4 mL of soft agar (tryptone soya broth containing 0.6% agar; Oxoid Ltd., Basingstoke, UK). Then, the mixture was spread onto a freshly prepared tryptone soya agar plate (containing 1.5% agar; Sigma-Aldrich, St. Louis, USA). This method was performed independently with each of the 22 referenced host strains for each filtrate. Overlay plates were incubated for 24 h at 30 °C, followed by phage purification [17].
- **b) Isolation after enrichment method:** A 10 g sample of silage or manure was mixed with 90 mL of modified Scholten’s (MS) broth (Condalab, Madrid, Spain) in a sterile Whirl–Pak bag with a filtered screen. Then, 250 µL of the overnight cultures for the 22 host strains were added singularly, and the cultures were adjusted to approximately 2.5 × 10^8^ CFU/mL using a spectrophotometer (Automatic Elisa Plate Analyser, Robonik, India). Samples were incubated at 30 °C for 24 h. An aliquot (100 µL) of each enriched sample was used for sequential filtration and phage isolation by the double-layer plate method, using the same method described above for direct isolation.

### Purification and propagation of phage titer

Single plaque from each plate was carefully removed from the agar using a sterile loop and resuspended in PBS (100 µL). Then, tenfold serial dilutions of plaque-PBS suspension were prepared. Overlay plates were prepared from four tenfold serial dilutions using the double-layer method described above, and this step was performed three times to obtain pure phages [19]. The isolated plaque from the third passage was suspended in 5 mL of PBS. After 24 h of incubation at 30 °C, 1 mL of chloroform was added, followed by centrifugation at 4,200 × *g* for 15 min and filtration of the supernatant using a 0.45-µm filter [14]. Then, the phage suspension was filtered (0.2 µm) and the previous steps were repeated until the phage titer reached 10^9^–10^10^ plaque forming units (PFU)/mL [20]. Phage stocks were stored at 4 °C.

### Characterization by transmission electron microscopy

One drop of each purified sample with a high phage titer (10^10^ PFU/mL) was deposited onto Formvar film copper grids coated with 200-mesh carbon (Electron Microscopy Sciences, Hitachi H600A, Japan) and stained using 1% uranyl acetate (pH 7.4). Samples were imaged at 73000 magnification power using a JEOL 1400 Flash transmission electron microscope at 120 kV (Talos L 120C G2-TEM- Thermofisher- UK).

### Host ranges of the isolated LMPs

Six LMPs that were isolated from silage and manure samples were tested against 22 *L. monocytogenes* strains with different ERIC sequences. The strains selected for the analysis represented 22 different ERIC types previously isolated from three dairy cattle farms (Supplementary Table 1), as described previously [21]. *L. monocytogenes ERIC B* was the most susceptible strain to the isolated LMPs, so it was employed as the control strain. A high-titer of each isolated phage (10^10^ PFU/mL) was spotted against each *L. monocytogenes* strain using double layer agar technique. Briefly, 100 μl of an overnight culture of *L. monocytogenes* strain was mixed with 3 ml of molten soft agar (0.4 percent) in a sterile tube which was poured on the surface of Muller-Hinton agar (MHA) (Oxoid Ltd., Basingstoke, UK) plate (1 percent). The plate was left on the bench until the soft agar has solidified then poured 5 μl of phage over the solidified soft agar. The plate was inverted and incubated at 37 °C overnight after they had absorbed all of the liquid. The plate was examined the next day for clearing zones.

### One-step growth curve

The one-step growth experiment was performed for LMP3 (the most infective phage) [22]. The *L. monocytogenes* strain (ERIC A) that was the most resistant to the isolated phages infection was cultured in 1 mL of BHI broth until an OD_600_ of 0.5 which corresponded to 1.5 × 10^8^ CFU was reached and then collected by centrifugation. The cells were resuspended with 0.9 mL of fresh BHI broth and mixed with 0.1 mL of LMP3 (the most effective phage) solution (1 × 10^8^ PFU/mL). The phages were allowed to absorb for 5 min, and the solutions were centrifuged at 13,000 × *g* for 1 min to remove any free phage particles. After discarding the supernatant, the phage-infected bacterial pellets were resuspended in 50 mL of prewarmed MS broth, and the cultures were incubated at 30 °C. Samples were harvested at intervals of 5 min, and phage titers were immediately determined by the double-layer agar method. The burst size of phages was calculated by dividing the final titer of released phage particles by the initial count of infected bacterial cells. This experiment was performed in triplicate.

### PH and thermal stability assays

For the pH stability tests, 100 μL of phage suspension (1.0 × 10^7^ PFU/mL) was used to inoculate 900 μL of physiological saline adjusted to pH values of 2.5, 3, 4, 7, 8, 9 and 12 using NaOH or HCl. The mixtures were incubated at 37 °C for 1 h and aliquots were taken to measure the titers of phages at different pH values. For thermal stability tests, 2 mL of phage suspension (1.0 × 10^7^ PFU/mL) was incubated at 4 °C, 25 °C, 30 °C, 40 °C, 50 °C, and 60 °C at pH 7.0. At intervals of 1 h, up to 4 h, 100-μL aliquots were collected. Survived phages were counted and the survival rates were calculated by the PFU at each time point divided by initial concentration of phage (1.0 × 10^7^ PFU). All tests were performed in triplicate [23].

### In vitro efficacy of isolated LMP alone, silver nanoparticles alone (AgNPs), and LMP-AgNPs composite against multidrug-resistant *L. monocytogenes* strains

The most effective phage (LMP3) and the most phage-resistant *L. monocytogenes* strain (ERIC A) were used for this study.

- **a) Silver nanoparticles** were purchased from nanoComposix (San Diego, CA, USA). AgNPs was prepared at final concentration of 10 µg/ml [10].
- **b) LMP3-AgNPs composite preparation:** The LMP3-AgNPs composites were prepared according to the procedures described previously [24]. In brief, phages (5 × 10^11^ PFU/mL) were incubated with AgNPs at a final concentration of 10 µg/mL [10] and a 1:4 ratio at 30 °C overnight with orbital shaking (KS130 Basic IKA) at 320 rpm. Then, the LMP3-AgNPs networks were purified by centrifugation at 20,800 × *g* for 30 min for the separation of free silver and unbound phages. The composites were resuspended in 5 mL of buffer and stored at 4 °C.
- **c) Host cell lysis test (time-kill curve)**
- The bactericidal effects of LMP3 alone, AgNPs alone, and the LMP3-AgNPs composite against *L. monocytogenes* (ERIC A) was determined by measuring the viable bacterial counts via detection of the optical density at 600 nm. The test was conducted in 24-well ELISA plates. *L. monocytogenes* suspensions were adjusted to obtain a final concentration of 8.0 × 10^6^ CFU/mL using a spectrophotometer (Automatic Elisa Plate Analyser, Robonik, India) (OD_600_ = 0.1), then 1.8 mL of suspension was distributed in each well [25]. Two hundred microliters of isolated LMP3 were added to each well to achieve a final concentration of 10^5^–10^7^ PFU/mL. The indicated multiplicities of infection (MOI: the ratio of phage to their host cell) were 0.1, 1, and 10. BHI broth (0.2 mL) without phages was added to the bacteria control well to represent the negative control in this test. At time zero (T 0 h), the *L. monocytogenes* and phage titers were confirmed. The plates were incubated at 30 °C, and the OD_600_ was read every 2 h for 24 h. The experiments were carried out in triplicate [26].

### Statistical analysis

Data were recorded in Microsoft Excel (version 15.0), and statistical analysis was carried out using Statistical Package for the Social Sciences software version 22. Descriptive statistics, including percentages and frequency distribution, were used, and the probability level (*P*) was calculated.

## Results

### *L. monocytogenes* phage isolation

A total of 36 silage and 36 manure samples (15, 9, and 12 samples from farms I, II, and III, respectively) were examined for LMPs. Of these, six samples were positive for phages, and six LMPs were recovered using 22 diverse *L. monocytogenes* hosts and two phage isolation methods (direct isolation and isolation after enrichment) (Table 1). All samples from farm I were LMP-negative for both methods. For farm II, four out of nine samples were positive for LMPs; three samples (one manure and two silage) were positive by enrichment only, and one silage sample was positive only by direct isolation method, which yielded four phage isolates. Meanwhile for farm III, 2 (silage) out of 12 samples were positive for LMPs after enrichment method only, yielding two phage isolates.

**Table 1:** Recovery of listeriaphages from manure and silage samples collected from dairy cattle farms

Type of sample	Manure			Silage		
Farm	No. of examined samples	No. of samples that yielded plaques		No. of examined samples	No. of samples that yielded Plaques	
		By Direct Isolation	By Enrichment Method		By Direct Isolation	By Enrichment Method
Farm I	15	0	0	15	0	0
Farm II	9	0	1	9	1	2
Farm III	12	0	1	12	0	1
Total	36	0	2	36	1	3

### Transmission electron microscope characterization of isolated LMPs

**Fig. 1:**
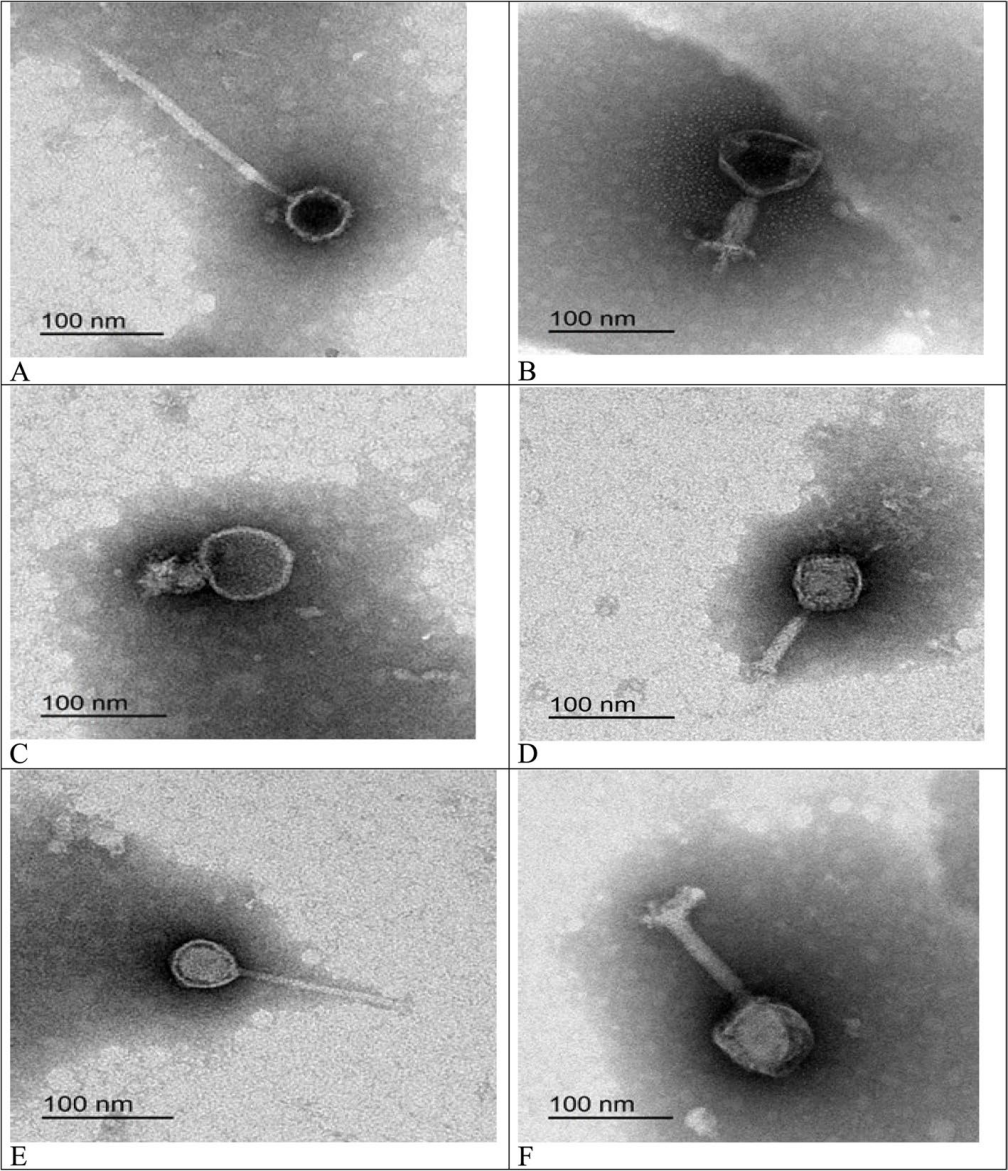
Transmission electron microscopy photograph of isolated *Listeria monocytogenes* phages (LMP). **A**: LMP1 and **E**: LMP5; are belonging to the *Siphoviridae* family. **B**: LMP2, D: LMP4 and **F**: LMP6; are belonging to *Myoviridae* family. **C**: LMP3; located in the *Podooviridae* family

Transmission electron microscopy (TEM) was used for the phenotypic characterization of the isolated LMPs (Table 2) (Fig. 1). LMP1 had an icosahedral-isometric head of 52.90 × 54.79 nm and a thin, long, non-contractile, flexible tail that was 223.77 nm in length; therefore, it was identified as a *Siphoviridae* family virus (Fig. 1; A). LMP2 had an icosahedral-isometric head of 77.11 × 82.48 nm and a thick, short, contractile tail that was 62.87 nm in length; therefore it was identified as a *Myoviridae* family virus (Fig. 1; B). LMP3 had an icosahedral head of 60.12 × 36.27 nm and a thick, very short, contractile tail that was 49.86 nm in length; therefore, it was identified as a *Podoviridae* family virus (Fig. 1; C). LMP4 had an icosahedral head of 54.95 × 60.04 nm and a thick, relatively short, contractile tail that was 64.65 nm in length; therefore, it was identified as a *Myoviridae* family virus (Fig. 1; D). LMP5 had an elongated head of 49.32 × 51.82 nm and a thin, long, non-contractile, flexible tail that was 162.03 nm in length; therefore, it was identified as a *Siphoviridae* family virus (Fig. 1; E). LMP6 had an icosahedral head of 83.05 × 74.87 nm and a thick, relatively long, non-contractile tail that was 99.42 nm in length; therefore, it was identified as a *Myoviridae* family virus (Fig. 1; F).

**Table 2:** Morphological characteristics of isolated *Listeria monocytogenes* phages (LMPs)

Phages	Capsid Diameter (nm)	Tail length (nm)	Tail width (nm)
LMP1	52.90 × 54.79	223.77	11.12
LMP2	77.11 × 82.48	62.42	23.57
LMP3	60.12 × 36.27	49.86	24.21
LMP4	54.95 × 60.04	64.65	13.70
LMP5	49.32 × 51.82	162.03	10.29
LMP6	83.05 × 74.84	99.42	15.13

### Host ranges of the isolated *Listeria monocytogenes* phages

The diversity of the six isolated *Listeria* phages (LMP1–LMP6) was determined through the detection of their host ranges against the 22 *L. monocytogenes* strains via plaque assay. We found that 14 (63.6%) of these strains were susceptible to LMP3 and that LMP2 could infect 10 (45.5%) strains. LMP4 had the ability to infect 9 (40.9%) strains. LMP5 and LMP6 were shown to be capable of infecting 8 (36.4%) of the 22 tested strains. LMP1 could infect only 7 (31.8%) of the tested strains. *L. monocytogenes* ERIC A was resistant to all isolated LMPs except LMP3 (Table 3).

**Table 3:** Host range of the isolated 6 LMPs for 22 multidrug-resistant *L. monocytogenes* from dairy cattle farms

Phage	LMP1	LMP2	LMP3	LMP4	LMP5	LMP6	No. of phages lysing specific strain
Listeria monocytogenes strains							
ERIC A	-	-	+	-	-	-	1
ERIC B	+	+	+	+	+	-	5
ERIC C	+	-	-	-	-	+	2
ERIC D	-	-	-	+	+	-	2
ERIC E	-	+	-	-	+	-	2
ERIC F	-	+	+	-	-	+	3
ERIC G	-	-	+	-	+	-	2
ERIC H	-	+	-	-	+	+	3
ERIC I	-	-	+	+	+	-	3
ERIC J	-	+	-	-	-	+	2
ERIC K	+		-	+	+	-	3
ERIC L	+	+	+	-	-	-	3
ERIC M	-	-	+	+	-	-	2
ERIC N	-	-	+	-	-	+	2
ERIC O	-	+	+	+	-	-	3
ERIC P	+	-	+	-	-	-	2
ERIC Q	-	-	+	+	-	+	3
ERIC R	-	+	-	-	-	+	2
ERIC S	-	-	+	-	+	-	2
ERIC T	+	+	+	-	-	-	4
ERIC U	-	-	-	+	-	+	2
ERIC V	+	+	+	+	-	-	4
Total No. of positive Hosts (%)	7 (31.8)	10 (45.5)	14 (63.6)	9 (40.9)	8 (36.4)	8 (36.4)	

#### One-step growth curve

**Fig. 2:**
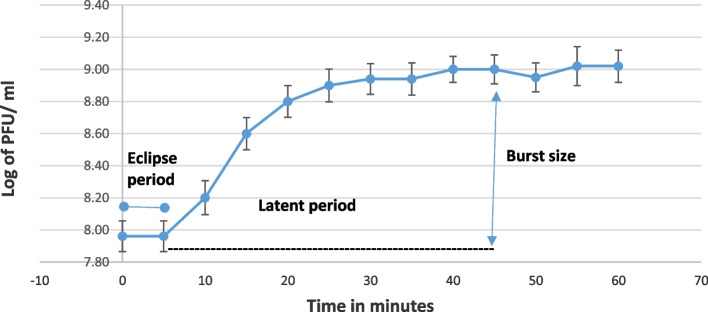
One step growth curve of LMP3. Eclipse, latent period and burst size of LMP3 at MOI of 0.1 and *L. monocytogenes* (ERIC A) adjusted at OD600 of 0.5

A one-step growth curve of LMP3 propagated on *L. monocytogenes* ERIC A was constructed three times to determine the latent time and phage burst size. The results were shown in Fig. 2. After 5 min of incubation, 92% of the phage particles adsorbed to the bacterial host cell. Eclipse and latent periods were observed at 5 and 45 min, respectively, and were followed by a short growth period of 10 min. The burst size of LMP3 was 25 PFU per infected cell.

#### pH and thermal stability

**Fig. 3:**
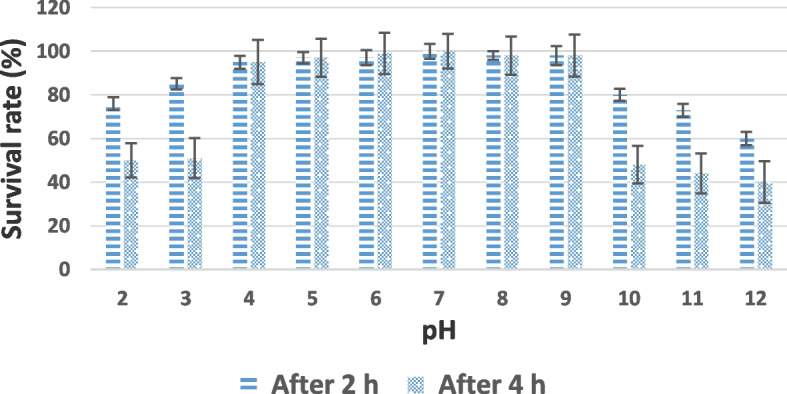
pH stability of LMP3. Phage was incubated for 4 h under different pH values, and the survival rate of phage were shown at 2 h and 4 h from the triplicate experiments

The pH and thermal stabilities of LMP3 were estimated by determining the changes in survival based on the number of PFU. As shown in Fig. 3, the growth of LMP3 showed no obvious change after 2 h and 4 h incubation with pH 4.0–9.0 at which the survival rate was ranged from 95.70% to 100%. The survival rate of LMP3 after 2 h incubation with increasing the alkalinity at pH 10.0, 11.0 and 12.0 was 80.60%, 72.70% and 60%, respectively, meanwhile after 4 h the lowest recovery of LMP3 was recorded to be 47.8%, 44.4% and 39.8%, respectively (Fig. 3). Also, the survival rate after 2 h with pH 2.0 – 4.0 was 75.8% and 84.6%, respectively which remain in the decreasing after 4 h to be 49.9% and 50.7%, respectively.

**Fig. 4:**
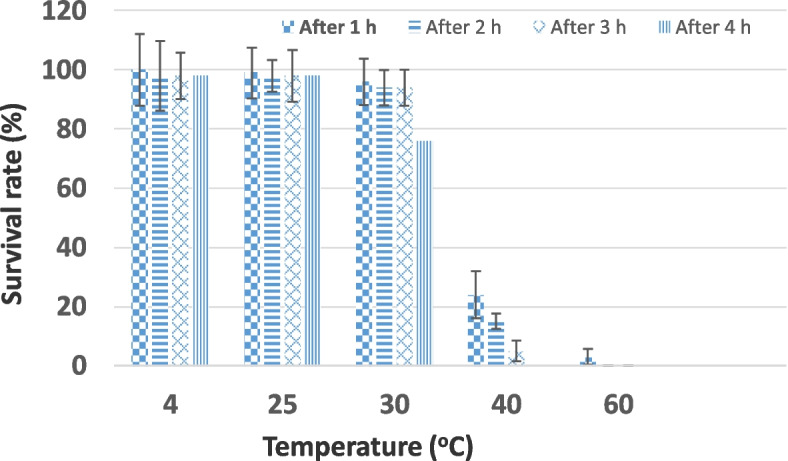
Thermal stability of LMP3. Phage was incubated for 4 h under different degrees of temperature (4, 25, 30, 40, 60 °C) and the survival rate of phage were shown at 1 h, 2 h, 3 h and 4 h from the triplicate experiments

The thermal stability of the LMP3 was detected at pH 7.0. Figure 4 showed that the highest survival rate (100% to 97.8%) of LMP3 after incubation at 4 °C and 25 °C for 4 h. **LMP3** relatively stable at 30 °C for 3 h only at which the recovery of phage reached to 93.8%. Meanwhile, LMP3 was sensitive to higher temperatures at which 24% and 3% of phage only remained alive after 1 h incubation at 40 °C and 60 °C, respectively. Also, no phages survived at 40 °C and 60 °C, after 4 h and 2 h, respectively.

### Host cell lysis (time-kill curve)

**Fig. 5:**
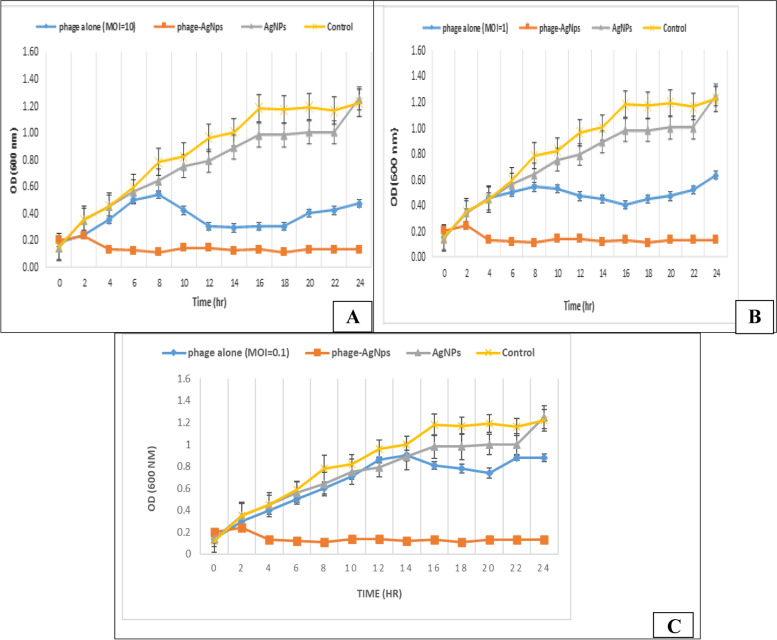
The Time–Killing curve of Multidrug resistant ***L. monocytogenes (ERIC A)*** with mixture of AgNPs of final concentration of 10 μg/mL and phage with multiplicity of infection (MOI) of 0.1 (**A**, **B** and **C**); with phage alone MOI 10 (**A**), MOI 1 (**B**) and MOI 0.1 (**C**) and with AgNPs alone of final concentration of 10 μg/mL (**A**, **B** and **C**)

The in vitro efficacy of isolated LMP3 alone, AgNPs alone and the LMP3-AgNPs composite against multidrug-resistant *L. monocytogenes* (ERIC A) was measured and represented in a time-kill curve to determine the differences in the optical density (OD_600_) changes. Spectrometric method was used for the detection of bacterial cells number through the measuring of OD at 600 nm, as it is rapid. LMP3 at an MOI of 0.1 in conjugation with 10 µg/mL of AgNPs had the ability to inhibit bacterial growth after 4 h of treatment by 0.32 OD_600_ compared with the control, and this effect persisted even after 24 h by reducing the intensity of *L. monocytogenes* to 1.09 OD_600_ (*p* < 0.001) (Fig. 5; A, B, and C). The data showed that LMP3 alone at an MOI of 10 was able to decrease the OD_600_ to 0.40 at 10 h, which persisted for 18 h of treatment (Fig. 5; A). In addition, LMP3 alone at the MOIs of 1 and 0.1 was able to reduce the bacterial growth by an OD_600_ of 0.43 and 0.37 (*p* < 0.001) at 12 h and 16 h of the experiment, respectively (Fig. 5; B and C). Meanwhile, the bacterial growth in the presence of AgNPs alone continued to increase for 16 h then remained constant for just 6 h and reincreased for 2 h until reached to the peak at 24 h from the beginning of the experiment (Fig. 5; A, B, and C). The most important finding was that the bacterial persistence occurred when the phage was used alone, but it was defeated when the phage was combined with AgNPs. Overall, the network of LMP3 (MOI of 0.1) and AgNPs at a sublethal concentration of 10 g/mL had a significant inhibitory effect when compared to using the phage alone at the same MOI (0.1) or even at an increased MOI (10).

## Discussion

The exponential increase and ascendancy of multidrug-resistant *L. monocytogenes* strains is a concern not only for dairy cattle farms but also for human health. This hazard can be efficiently controlled by the use of bacteriophages and silver nanoparticles. In this study, six different LMPs were recovered. Frequent isolation of LMPs occurred from silage samples obtained from dairy farm II (three out of nine samples) using 22 *L. monocytogenes* strains. Five of these phages were recovered by the enrichment method [17]. The enrichment method is a simple and efficient method for the isolation of scarce phages from the environment and very suitable for the isolation of a few phages for particular hosts [27]. Meanwhile, Vongkamjan et al. [17] detected the phages by direct isolation not by enrichment method and attributed it to degradation of phages during enrichment due to proteases or nucleases present in the enrichment MS broth. No LMPs were isolated from manure or silage samples from farm I, which may be attributed to a high level of hygiene and the frequent removal of manure. Bacteriophages are common and reliable indicators of bacterial contamination because their occurrence is high in surroundings where their hosts are numerous [28]. The high occurrence of LMPs in silage samples shows that inadequately fermented silage, as well as dairy farm environments in general, are suitable substrates for LMP isolation. In most cases, phages were found in moist samples with a high pH and low lactic acid production. In silage with a pH of 4.1 or below, no phages were detected [29].

The classification of isolated phages was based on the morphology observed under TEM, which plays a key role in phage characterization [28, 30]. The classification was based on the characteristics of the head and the tail of the phage. If a tail is present, it can be contractile or non-contractile and short or long in comparison to the head size. Long-tailed bacteriophages have tails that are longer than the head diameter, while short-tailed phages have tails that are shorter than the diameter of the head [31]. This study revealed that the six isolated LMPs had different morphological characteristics and were consequently classified into three groups. The first group included LMP1 and LMP5, which had long, flexible tails and belonged to the *Siphoviridae* family. The second group included LMPs 2, 4, and 6 and had contracted tails and icosahedral heads, which are characteristic of the *Myoviridae* family. The third group included LMP3, which had a very short tail and was identified as a *Podoviridae* family virus. These findings were consistent with prior studies on the morphological properties of phages [32]. In the study by Song et al. [33], all isolated phages were classified within the *Siphoviridae.* The *Listeria* phages discovered so far belong to the order *Caudovirales*, which contains the *Siphoviridae* family as a prominent subgroup [34]. However, no phages in the *Podoviridae* family were stated in the recent study conducted by Ribeiro et al. [35]. LMP3 had the shortest tail and was the most infective phage. The length of the tail provides an insight into the phage stability and resistance in the wild; short and non-tailed phages are more resistant, while lengthy tails are more easily destroyed, resulting in phage infectivity loss [28].

The host range of a phage has various implications for its utility in phage treatment. A single-species host range is preferable since it stops the phage from killing other species, therefore keeping the remainder of the host's microbiome unharmed [36]. In phage treatment, using phages with a greater host range would theoretically result in fewer treatment failures due to a mismatched host and phage combination; hence a broader host range is desirable in terms of strains within target species [37].

In our study, we isolated six LMPs from two dairy cattle farms and tested the host range of these phages against 22 different ERIC types of *L. monocytogenes*. A bacteriophage's host-specific lytic activity against its host bacterium is largely reliant on connection between the phage's tail fiber and receptor components on the bacterial cell wall [38]. The host range tests revealed that all 22 (100%)* L. monocytogenes* strains were susceptible to phage infection. Fifty percent (3 out of 6) of the isolated phages showed a narrow host range, and 50% (3 out of 6) of phages showed a moderate host range at which the LMP3 is the most infective one. Wide host range phages were not detected in the present work. In the study which conducted by Vongkamjan et al. [39], broad-host-range phages infected 84.6% of the tested *L. monocytogenes* strains, while restricted-host-range phages infected 7.7% to 38.5% of the *L. monocytogenes* strains. *Listeria* phages with wide host ranges have been previously identified [39–41], and infect a greater range of *L. monocytogenes* serotypes than LMPs with small host ranges [42, 43]. It is worth noting that phages only infect specific bacterial hosts. The fact that phage treatment can focus precisely on the disease without affecting commensal bacterial flora is often seen as a benefit over standard antibiotics [44]. However, bacteria develop resistance to phages quickly; therefore, the antibacterial impact gained may be transitory [45, 46].

The LMP3 was found to have a short adsorption time, in which 92% of LMP3 adsorbed to *L. monocytogenes* (ERIC A) in 5 min. This is in contrast with LP-018 in which 60 min was required for adsorption of 85.1% [33], LP-027 and LP-094, in which 30 min was required to attain adsorptions of 96.4% and 97.8%, respectively [47], also LP-048 and LP-125 have longer adsorption time (20 min) [48]. The latent period of LP-027 and LP-094 was about 45 min [49] which is similar to LMP3. The eclipse period of LP-018 was between 60 and 90 min, the eclipse period of LP-048 and LP-125 were 40–50 min and 35–40 min, respectively, which is much longer than the LMP3. The calculated burst size of LMP3 was much higher than LP-018 (approximately 2 PFU/cell) [33], LP-048 (13.6 PFU/cell) and LP-125 (21.3 PFU/cell) [48]. On the other hand, burst size of LMP3 slightly higher than LP-027 and LP-094 which was 34 and 28, respectively.

When LMP3 was subjected to various different conditions of pH (4–10), the data clarified that the phages can remain active under a wide range of pH conditions but sensitive to strong acid or alkali specially with increasing the incubation period more than 2 h. When the effect of heat was evaluated on LMP3 it was observed that LMP3 was sensitive to heat at higher temperature, this finding was in harmony with that found by Vinodkumar et al. [49] and Liu et al. [23].

Time-kill curves were constructed to illustrate the in vitro efficacy of isolated LMP3 (the most infective one to the bacterial strains) alone and the LMP3-AgNPs composite against multidrug-resistant *L. monocytogenes* (ERIC A). The curves showed that the combination of AgNPs (10 g/mL) and LMP3 at an MOI of 0.1 had the highest effect in reducing *L. monocytogenes* growth (maintenance effect), without any secondary bacterial growth, followed by LMP3 alone at an MOI of 10. The treatment with LMP3 alone at an MOI of 0.1 had the lowest effect. AgNPs (10 g/mL) alone has the lowest inhibition rate comparing with the other treats. However, secondary bacterial growth due to bacterial resistance was observed with the administration of phages alone at the MOIs of 0.1, 1, and 10. We propose that by combining phages with AgNPs, we can determine their biocontrol capacities using low concentrations of AgNPs and phages without creating resistance.

The results demonstrated significant differences (*p* < 0.001) between the use of phages alone with different MOIs (0.1, 1, and 10) and the use of phages at an MOI of 0.1 in combination with AgNPs. An important observation was the stability of the antimicrobial effect of the *Listeria* phages (0.1 MOI) in combination with AgNPs (10 µg/mL), which did not occur when phages were used alone, even at an MOI of 10. Since preceding research has indicated that large doses of AgNPs inactivate T4 phages, these stability findings cannot be applied to all phage families [50]. The synergistic action between *Listeria* phages and AgNPs, with minimum doses for both, against multidrug-resistant *L. monocytogenes* will consequently reduce the development of resistance to phages and limit the side effects of AgNPs on animals and their environments. From a pharmacokinetic standpoint, low concentrations of phage will reduce the likelihood of it being identified by the immune system and cleared from the human/animal body [51]. Our experiment carried out for achieving preliminary data. Therefore, before the potential use of *Listeria* phages and AgNPs as antibacterial agent, the full genetic characterization of the phage will be performed. In particular, the safety of the phages should be assessed (e.g., verification of the strictly lytic cycle, absence of genes coding for anti-microbial resistance, absence of toxin genes).

## Conclusions

This is the first report for the biocontrol of multidrug-resistant *L. monocytogenes* using phages alone and in combination with AgNPs. In this study, six different phenotypic *L. monocytogenes* phages (LMP1–LMP6) were isolated from silage and manure from dairy cattle farms. These phages belonged to three different families: *Siphoviridae*, *Myoviridae*, and *Podoviridae*. LMP3 was isolated with the most of host strains, low latent periods, and wide pH and thermal tolerance. Also, LMP3 at an MOI of 0.1 in combination with AgNPs (10 µg/mL) exhibited complete inhibition activity against *L. monocytogenes* strains after 6 h, and the antibacterial effects lasted for the whole 24 h of the treatment. In comparison to the other treatments, the combination of AgNPs and LMP3 exhibited a considerable reduction in *L. monocytogenes* (OD_600_) growth and good stability of action. These findings suggest the use of phage-silver nanoparticles as an advanced biocontrol approach for combating multidrug-resistant *L. monocytogenes* in dairy cattle farms.

## Supplementary Information

**Additional file 1:** **Supplementary Table 1.** Listeria monocytogenes strains used in this study.

**Additional file 2.** 

**Additional file 3.** 

## Data Availability

All data generated or analyzed during this study are included in this published article and its supplementary information files.

## Abbreviations

AgNPs: Silver nanoparticles
BHI: Brain Heart Infusion
ERIC: Enterobacterial Repetitive Intergenic Consensus
*L. monocytogenes*: *Listeria monocytogenes*
LMP: Listeria monocytogenes phage
MHA: Muller Hinton agar
OD: Optical density
PFU: Plaque Forming Unit
TEM: Transmission electron microscope
PBS: Phosphate-buffered saline
MOIs: Multiplicity of Infections
