# Supplementary material for: Isolation and characterization of bacteriophages for combating multidrug-resistant Listeria monocytogenes from dairy cattle farms in conjugation with silver nanoparticles
Source: BMC Microbiol. 2023 May 22;23:146. doi: 10.1186/s12866-023-02893-y (PMC10201749; doi:10.1186/s12866-023-02893-y)
Supplement: Supplementary file 1 — Additional file 1: Supplementary Table 1. Listeria monocytogenes strains used in this study. [file 12866_2023_2893_MOESM1_ESM.docx]

**Supplementary:**

**Table 1. *Listeria monocytogenes* strains used in this study**

| Antibiotics resistance genes | Virulence genes | *Antibiotics pattern | ERIC-PCR Type | Isolates No.  Or ID | Type of sample | Source | Farm |
| --- | --- | --- | --- | --- | --- | --- | --- |
|  | | | | | Milk | Animals | Farm II |
| CTX-M, DHA, *qnrA,qnrB,qnrS,parC, ermB, msrA, dfrD, tetM, int-Tn* | *prfA* | 24 | M | 59 |  |  |  |
| CTX-M, DHA, *qnrA,qnrB,qnrS,parC,ermB, msrA, dfrD, tetM* | *hlyA, prfA* | 25 | N | 51 |  |  |  |
| CTX-M, DHA, *qnrA,qnrS,parC,ermB, dfrD, tetM, int-Tn* | *hlyA, prfA* | 25 | Q | 45 |  |  |  |
| CTX-M, DHA, *qnrA,qnrB,qnrS,parC, ermB, tetM* | *hlyA, prfA* | 19 | S | 66 |  |  |  |
| CTX-M, DHA, *qnrA,qnrS,parC, ermB, dfrD, tetM* | *hlyA, prfA* | 25 | T | 47 |  |  |  |
| CTX-M, DHA, *qnrA,qnrB,qnrS,parC, ermB , msrA, dfrD, tetM* | *hlyA, prfA* | 25 | U | 44 |  |  |  |
|  |  |  |  |  | Feces | Environment |  |
| CTX-M, DHA, *qnrA,qnrB,qnrS,parC, ermB, msrA, dfrD, tetM, int-Tn* | *hlyA, prfA* | 24 | A | 94 |  |  |  |
| CTX-M, *qnrA,qnrB,qnrS, parC, ermB, msrA, dfrD, tetM, int-Tn* | *hlyA, prfA* | 24 | F | 31 |  |  |  |
|  |  |  |  |  |  |  |  |
| CTX-M,DHA, qnrS, ermB, msrA, dfrD, tetM, int-Tn | *hlyA, prfA* | 16 | I | 79 |  |  |  |
| CTX-M, DHA, *qnrA, qnrB, qnrS, parC, ermB, msrA, dfrD, tetM, int-Tn* | *hlyA, prfA* | 25 | J | 43 |  |  |  |
| CTX-M, DHA, *qnrA,qnrB,qnrS, parC, ermB, msrA, dfrD, tetM, int-Tn* | *hlyA, prfA* | 24 | G | 58 |  |  |  |
|  |  |  |  |  |  |  |  |
|  |  |  |  |  | Silage |  |  |
| CTX-M, *qnrA,qnrB,qnrS,parC ermB, dfrD, tetM, int-Tn* | *hlyA, prfA* | 24 | C | 61 |  |  |  |
|  | | | | |  | Milking equipment’s |  |
| CTX-M, *dfrD, tetM* | *hlyA, prfA* | 25 | E | 48 | Teat cups swab |  |  |
|  |  |  |  |  |  |  |  |
| CTX-M, DHA, *qnrS, ermB, msrA, dfrD, tetM, int-Tn* | *hlyA, prfA* | 17 | K | 83 |  |  |  |
| CTX-M, *qnrS, ermB, dfrD* | *hlyA, prfA* | 12 | H | 85 | Floor swabs |  |  |
|  |  |  |  |  |  |  |  |
|  |  |  |  |  | Milk | Animals | Farm III |
| CTX-M, DHA, *qnrA,qnrB,qnrS,parC, ermB* | *hlyA* | 24 | L | 96 |  |  |  |
| CTX-M, DHA, *qnrS, ermB , msrA, tetM, int-Tn* | *hlyA, prfA* | 13 | O | 135 |  |  |  |
| CTX-M, DHA, *qnrA,qnrB,qnrS,parC, ermB, dfrD,tetM* | *hlyA* | 24 | P | 101 |  |  |  |
| CTX-M, DHA, *qnrS, ermB, msrA, dfrD, tetM, int-Tn* | *hlyA* | 22 | R | 102 |  |  |  |
|  | | | | |  |  |  |
| CTX-M, DHA, *qnrS, ermB, dfrD, tetS* | *hlyA* | 22 | D | 106 |  |  |  |
|  | | | | |  |  |  |
|  |  |  |  |  |  | Environment |  |
| CTX-M, *qnrS, ermC, tetS* | *hlyA,prfA* | 13 | B | 134 | Silage |  |  |
|  | | | | |  |  |  |
|  |  |  |  |  | Manure |  |  |
| CTX-M, DHA, *qnrS, ermB, dfrD, tetM* | *hlyA, prfA* | 10 | V | 126 |  |  |  |
|  |  |  |  |  |  |  |  |

**The reference of the strains is [5].**
